# Editorial: The impact of climate change on nutrient composition of staple foods and the role of diversification in increasing food system resilience

**DOI:** 10.3389/fpls.2023.1087712

**Published:** 2023-01-23

**Authors:** Carla S. Santos, Ephrem Habyarimana, Marta W. Vasconcelos

**Affiliations:** ^1^ Universidade Católica Portuguesa, CBQF - Centro de Biotecnologia e Química Fina – Laboratório Associado, Escola Superior de Biotecnologia, Porto, Portugal; ^2^ Sorghum Breeding, International Crops Research Institute for the Semi-Arid Tropics (ICRISAT), Patancheru, Telangana, India

**Keywords:** elevated CO_2_, environment - agriculture, food security, intercropping, landraces, nitrogen metabolism

A staple food crop is a crop that makes up the dominant part of a population’s diet, being the primary source of energy and nutrition. The Food and Agriculture Organization (FAO) reported that maize, rice and wheat provide about 60 per cent of the world’s food energy intake. Soybean (an oil crop) is the most commonly used crop in animal feed since it has high protein content. Given the high demand for these four crops, several agricultural technologies and practices have focused on increasing their productivity and adaptation to different environmental conditions (HarvestPlus, 2021). Specific interventions include increased technological investment, biofortification programs, machinery and selective agrochemicals, and advanced agronomic management practices.

However, agricultural conditions are rapidly changing due to current climate change events. These include increased greenhouse gas (GHG) concentrations in the atmosphere, global air temperature increase, drought events, sea surface temperature, and erratic precipitation patterns (Jones and Driscoll, 2022). These events harm crop production and setback the developed efforts to ensure a stable supply to the growing world’s population. Therefore, food security is at risk, and current food systems are under extreme pressure, which evidences the inequalities in food access and availability, access to land, and the working conditions for farm workers (Farooq et al., 2022). Adding on to food insecurity, undetermined consequences of climate change on agricultural production may also aggravate nutritional insecurity, which consists of the insufficient intake of essential nutritional components, such as vitamins, minerals, protein, or fat (Semba et al., 2022).

Current food systems are drivers of the climate change scenario. They contribute to about 35% of the global GHG emissions, biodiversity loss, and water pollution, all of which being highly unsustainable (Xu et al., 2021). A transition to a more climate-positive strategy in the agro-food sector is needed to include nutrient density and crop diversification, integrated with targeted policy actions and stakeholders’ capacity-building programs (Raven and Wackernagel, 2020; Iannetta et al., 2021). Well-founded research must support this transition, evidencing how future climate events will impact food safety and nutritional profile, given that current data mainly focuses on crop production in face of weather variables (Marmai et al., 2022). However, there are few recent studies that suggest that the increasing CO_2_ levels, heat, drought, or salinity stresses, either single or combined, alter nutrient allocation pathways and accumulation in staple crops, such as soybean and wheat (Soares et al., 2021; Zhara et al., 2022).

Huang et al. showed the importance of the interplay between N metabolism and C assimilation in rice. The increasing CO_2_ concentration in the atmosphere is expected to impact carbon (C) assimilation levels in plants. Additionally, one important player in environmental sustainability is nitrogen (N) and the lack of efficient N fertilization management in the last decades has resulted in increased GHG emissions, global warmth, and water resources eutrophication. Huang et al. research focused on understanding the role of *Oryza sativa DNA BINDING WITH ONE FINGER 11* (*OsDOF11*) transcription factor in nitrogen metabolism. The authors observed that *OsDOF11* induces sucrose transport-related genes and affects N assimilation, and mediates water and amino acids content. This fact might be of interest given the correlation between this activity with the ability of rice to cope with water deficit stress.

Fan et al. also investigated the role of N metabolism in wheat plants, under elevated CO_2_ conditions. For long, elevated CO_2_ conditions have been associated with decreased N and protein content, as well as other nutrients, in non-legumes and C_3_ crops e.g., rice and wheat. The mechanisms behind this effect are still elusive, although common knowledge appoints the ‘dilution effect’ as the main explanation for the nutritional decrease. Coherently, the authors observed that elevated CO_2_ induced N uptake, but the aboveground organs showed N deficiency, along with decreased concentration of potassium (K) and phosphorus (P). The authors considered that this study corroborated the dilution effect theory, where increased N uptake does not match the increase in carbohydrate synthesis. Fan et al. also considered that this might not be the only mechanism explaining the nutritional decrease, and the effect of elevated CO_2_ on NO_3_^-^ assimilation should be further studied. The impact of climate change on N acquisition may also impair other nutrient metabolisms, such as phosphorus (P), sulfur (S), zinc (Zn) and iron (Fe) (Kumar et al., 2021). Specifically, Fe and S are limiting factors in N acquisition since these two elements, in the form of the Fe-S cluster, are co-factors of enzymes of the reductive assimilatory pathway of N (Jiang et al., 2021). If this interplay is disrupted, plant growth, development and productivity processes may be affected, as well as the nutrient use efficiency, which may aggravate agricultural inputs use.

As alluded to in the title of the current Research Topic, crop diversification is one important measure to increase food system resilience, addressing food production challenges. There are different methods for achieving crop diversification in agricultural systems, namely, crop rotation, multiple cropping, or intercropping (Barman et al., 2022). These techniques bring important advantages to the soils and main crops, such as improved water use efficiency (Te et al., 2023) or ecosystem functions (Xiao et al., 2023); however, there may also be some disadvantages, such as the need for advance planning of planting, cultivation, fertilization, spraying, and harvesting, and ultimately identifying the best crop choices that reduce the likelihood of having to increase inputs.

Luo et al. analysed the impact of intercropping wheat with faba beans on N fertilizer requirements and photosynthetic mechanisms. The authors showed that intercropping had a positive effect on wheat growth parameters and yield, but this effect tended to be negatively correlated with the amount of N fertilization. Luo et al. suggest that the improved wheat biomass and yield in intercropped plants is a result of a better-structured canopy, that increased light energy utilization efficiency and optimized the light environment. Malhotra et al. also addressed the importance of crop diversification to improve biodiversity. Their research focused on the characterization of several traditional crops’ agro-morphological performance and physicochemical traits. The landraces collection, comprising 11 maize, seven paddy rice, three finger millet, five buckwheat, and three naked barley accessions, displayed phenotypic and genetic diversity, particularly in ear length, ear height, plant height, and 100-seed weight. Malhotra et al. also demonstrated that the nutritional and agro-morphological traits are associated with the geographic origin of the landraces. The authors pinpointed the role of promoting traditional crop landraces in germplasm conservation and agricultural systems sustainability and how this may address nutritional security.

Evidently, the above discussed mechanisms greatly impact soil health. The soil ability for C and N sequestration, or the transformation of P in plant rhizosphere, are highly dependent on the biodiversity of the microbial communities (Xu et al., 2022). Recent studies showed that elevated CO_2_ (Wang et al., 2023), changing precipitation patterns (Zuo et al., 2022), or fluctuations in temperature (Zhou et al., 2022), differently affect soil microbial communities. Therefore, soil microbiome, functionality and fertility should be considered as a complex network, and all players should be considered when looking at future scenarios.

This Research Topic compiled four papers addressing different consequences and rising mitigating challenges to address food quality and security in the context of climate change. The world population is expected to reach 9.7 billion by 2050, increasing the pressure on agriculture and the broader food system to deliver food, feed, and ecosystem services – all while mitigating the negative consequences of climate change. Agri-food systems actors and activities may help improve food production, processing, distribution, consumption, and disposal. The low agricultural diversity severely affects biodiversity and environmental sustainability, leading to soil degradation and higher global emissions. To achieve crop diversification and ensure food and nutritional security, current staple crops cultivation should be combined with underutilised crops, which are crops with great nutritional value and well-adapted to agro-climate niches (Singh et al., 2022). Therefore, it is essential to promote the cultivation of underutilised crops, and showcase their value compared to highly cultivated crops, which are nonetheless essential players in ensuring global food security.

## Author contributions

All authors listed have made a substantial, direct and intellectual contribution to the work and approved it for publication.
